# Self-organization of developing embryo using scale-invariant approach

**DOI:** 10.1186/1742-4682-8-17

**Published:** 2011-06-03

**Authors:** Ali Tiraihi, Mujtaba Tiraihi, Taki Tiraihi

**Affiliations:** 1 College of Computer and Electrical Engineering, Shaheed Behshti University, Tehran, Iran; 2 Department of Computer Engineering, Sharif University of Technology, Tehran, Iran; 3 Department of Anatomical Sciences, Faculty of Medical Sciences, Tarbiat Modares University, Tehran, Iran

## Abstract

**Background:**

Self-organization is a fundamental feature of living organisms at all hierarchical levels from molecule to organ. It has also been documented in developing embryos.

**Methods:**

In this study, a scale-invariant power law (SIPL) method has been used to study self-organization in developing embryos. The SIPL coefficient was calculated using a centro-axial skew symmetrical matrix (CSSM) generated by entering the components of the Cartesian coordinates; for each component, one CSSM was generated. A basic square matrix (BSM) was constructed and the determinant was calculated in order to estimate the SIPL coefficient. This was applied to developing *C. elegans *during early stages of embryogenesis. The power law property of the method was evaluated using the straight line and Koch curve and the results were consistent with fractal dimensions (fd). Diffusion-limited aggregation (DLA) was used to validate the SIPL method.

**Results and conclusion:**

The fractal dimensions of both the straight line and Koch curve showed consistency with the SIPL coefficients, which indicated the power law behavior of the SIPL method. The results showed that the ABp sublineage had a higher SIPL coefficient than EMS, indicating that ABp is more organized than EMS. The fd determined using DLA was higher in ABp than in EMS and its value was consistent with type 1 cluster formation, while that in EMS was consistent with type 2.

## Background

Self-organization is a property of the biological structure [1] and is reported to be important in protein folding [2-4]. It has also been documented that at higher hierarchical levels such as the organelle level, it has a crucial role in the biogenesis of secretory granules in the Golgi apparatus [5,6]. Martin and Russell have shown that self-organization exists in mitochondria, where redox reactions are localized [7]. The most obvious example of self-organization at the organelle level is the cytoskeleton during the mitotic cycle, where mitotic spindle forms dynamically [8] using molecular motors [9]. Misteli concluded that self-organization could govern the mechanistic principles of cellular architecture [10]. The multicellular embryo develops from a zygote, characterized by a dynamic self-organizing process [11].

At an early stage of embryonic development, the forming cells adhere to each other [12] with coordinated cellular movement to form the primary embryonic body axis [13]. These movements are self-regulated and lead to a defined pattern [14]. In vitro studies have confirmed self-organization in human embryonic stem cell (hESC) differentiation, resulting in the formation of the three germ layers and gastrulation [15]. Ungrin et al. reported a similar finding in the morphogenesis of hESCs cultured in suspension, which yielded embryoid bodies [16] with the property of self-organization. At later developmental stages such as organogenesis, Schiffmann reported self-organization in driving gastrulation and organ formation [17], where the increase in the mass of the organ and its cell number reportedly contribute to organogenesis [18]. Moreover, in vitro organogenesis showed a mechanism similar to that in vivo [19]. Among the factors contributing to organogenesis is self-organization; for example, in vitro organogenesis of the cultured mouse submandibular salivary gland at embryonic day 13 retains the capacity for branching, and when it is co-cultured with mesenchymal tissues, morphological differentiation of the gland results [20]. Similar results were obtained with cultured embryonic kidney explants leading to nephronal differentiation [21]. Other investigators introduced developmental self-organization in order to evaluate the morphogenesis of the embryo [22]. While the development of the embryo from a zygote to a multicellular organism is characterized by a dynamic self-organizing process [11], the emergence of an organized system is also associated with the expression of gene networks [23]. This could demonstrate the advantage of applying self-organization to cellular events [24]. In the present study, early development of *C. elegans *was investigated as an example of self-organization using a scale-invariant power law to evaluate the self-organizing properties of two sublineages with different differentiation fates.

Two features should be considered in the quantitative validation of a self-organization process in a developing embryo. First, the different scales of the animal body; for example, Waliszewski et al. reported that the microscopic gene expression and the macroscopic cellular proliferation were scale-invariant systems [25], the scale-free feature of which was shown to result in the emergence of organizational dynamics at all hierarchical levels of the living matter [26]. Secondly, metazoan cells develop from a single cell, and this involves complex spatio-temporal events [11,27].

Moreover, Molski and Konarski revealed that the fractal structure of the space in any biological system could characterize self-organization [28]. The fractal method can be used to describe the irregularity of shapes that cannot be formulated in Euclidean geometry. It is characterized by self-similarity [29], and describes spatial structure in a scale free measure [30]. In this study, the development of *C. elegans *embryos was evaluated at different time frames (stages) using a scale free power law. This method was developed in order to integrate spatial with temporal information. Moreover, the changes in the numbers and positions of cells during morphogenesis have been represented by the Cartesian coordinates at different developmental times. The components of the Cartesian coordinates were entered as the primary set data for calculating the power law coefficient in order to define the expanding character of the growing embryo.

## Materials and methods

### The concepts of CSSM and BSM

A matrix is an array of numbers arranged in rows (*i*) and columns (*j*). If the number of rows and columns is equal (*i*, *j *= *n*, *n *× *n*), then this matrix is a square matrix. The elements above the diagonal elements are considered as the upper triangular matrix of the square matrix and those below the diagonal elements are its lower triangular matrix. If the elements in the upper and lower triangular matrix of the matrix have equal values(*a_i,j _*= *a_j,i_*), then it is a symmetric square matrix. If all the elements in the upper triangular matrix have negative values of the lower triangular matrix or vice versa (*a_i,j _*= -*a_j,i_*), then it is a skewed symmetric (anti-symmetric) matrix, and if the diagonal elements values are equal to zero, the matrix is known as "zero centro-axial skewed-symmetric matrix" (CSSM). The matrix resulting from the exchange of the upper and lower triangular matrices is a transpose matrix. If the a square matrix is subtracted from its transpose, followed by division by two, then the resulting matrix is a skew matrix, while the sum of a square matrix and its transpose followed by division by two is a symmetric matrix. The square matrix is used for generating symmetric and anti-symmetric matrices. The square matrix generated in this study by a special algorithm is called the basic square matrix (BSM).

### The scale invariant power law

There are two aspects of the scale invariant power law: scale invariance means that the value of the SIPL coefficient does not change as the scale [31], magnification [32], or tissue growth changes [33,34]; and a power law is a relationship between two variables where one quantity varies as a function of the power of the other [33]. For example, Zhang and Sejnowski revealed that the growth of the volume of the white matter increases disproportionately more quickly than the gray matter, where it follows a power law relation [35]. In fact, one of the properties of power laws is scale-invariance [33]. Therefore, the SIPL defines the coefficient obtained by calculating the BSM determinant, which follows a power law rule and is scale invariant.

The reason for using the power law is the nature of the biological matter, over 21 orders of magnitude consistently follows a simple and systematic empirical power law. This includes metabolic rate, time scales and body size [36]. The most commonly used power laws are fractal dimension and allometry [37]. Fractal dimensions have been used to study diverse structures in nature at different levels and from galaxies [38] to subatomic structures [39]. In biomedicine, there are wide ranging applications; for example, at the molecular level, fractals were proposed for evaluating the physical features of ion channel proteins [40]. Vélez et al. reported the possible use of multifractals in the measurement of local variations in DNA sequence in order to define the structure-function relationship in chromosomes [41], and Mathur et al. used fractal analysis of gene expression in studying the hair growth cycle. Moreover [42], fractal genomics modeling has been used to predict new factors in signaling pathways and the networks operating in neurodegenerative disorders [43]. At the cellular level, fractal dimension was used in evaluating the morphological diversity of neurons and discriminating them on the basis of the neuronal extensions [44]; fractals can also explain higher orders of organization in biological materials such as the organization of tissues [45] and branching of tubular systems such as the respiratory and the vascular systems [46-49]. On the other hand, one of the best known applications of allometry is the metabolic rate scale (Kleiber's law), which is considered universal among different species, within the same species, or in individual animal at different orders including molecular, cellular and body levels [50,51]. A similarly universal allometric law relating time and body weight, including growth rates and animal age, has been documented [52]. This time scale relation is noticed in development biology [53]. Gillooly et al. reported an allometric relationship between metabolic rate and the developmental growth rate during embryogenesis, which has phylogenically and ontogenically invariant values [54]. Allometry was recently used in pharmacokinetics [55], predicting the pharmacokinetics of drugs [56,57]. In addition to allometry and Kleiber's law, other investigators have reported power law relationships in biomedicine; for example, Grandison and Morris reported that kinetic rate parameters showed a scale free relationship with the gene network and protein-protein interactions, which follows Benford's law [58]. Also, Zipf's Law has been used to discriminate the effect of natural selection from random genetic drift [59]; Furusawa and Kaneko (2003) reported that Zipf's Law applies universally to gene expression in yeast, nematodes, mammalian embryonic stem cells and human tissues [60].

The above discussion suggests that not every power law is fractal; on the other hand, in certain situations the behavior of the system shows fractal-like properties but is not truly fractal [61]. In addition, even natural fractal structures such as the triadic Koch curve could have non-fractal properties [62]. The growth of differentiating cells in a developing embryo certainly follows a power law, so we are justified in calling it SIPL to avoid fallacious attribution of fractal properties.

### Analytical descriptions of CSSM and BSM

#### CSSM

Suppose we have n points {(*x*_1_, *y*_1_, *z*_1_), (*x*_2_, *y*_2_, *z*_2_), ..., (*x*_*n*_, *y*_*n*_, *z*_*n*_)} ⊂ *R*^3 ^and relative 1 × n matrices {(*x*_1_, *x*_2_, ..., *x*_*n*_), (*y*_1_, *y*_2_, ..., *y*_*n*_), ..., (*z*_1_, *z*_2_, ..., *z*_*n*_)}. By subtracting the first entry *x*_1 _from *x*_*i*_, for each 1 ≤ i ≤ n, we get (0, *x*_2 _- *x*_1_, ..., *x*_*n *_- *x*_1_), with 0 as its first entry. We do this for the other matrices. Now we use each of the resulting matrices as the first row of the *CSSM *matrix. The other rows of the *CSSM *matrix are defined using the recursive formula:

We only need to prove that the matrix which is constructed from (*a*_1_, *a*_2_, ..., *a*_*n*_),

where *x_i,j_*= *a_j_*- *a_i_*, is anti-symmetric and hence it is *CSSM*. For each *i,j*

we have . Let *x_i,j _*= *a_j _*- *a_i_*. Then *x_i,j _*= -(*a_i _*- *a_j_*) = -*x_j,i_*, so the matrix is anti-symmetric. Now, we need to prove that when *x_i,j _*= *a_j_*- *a_i_*, then we have *x*_*i*+1,*j *_= *a_j _*- *a*_*i*+1_.

We have *x*_*i*+1,*j *_= *x_i,j _*- *x*_*i,i*+1 _= *a_j _*- *a_i _*- (*a*_*i*+1 _- *a_i_*) = *a_j _*- *a*_*i*+1_.

Also, by definition, this holds for the first row. So for every *i,j, x_i,j _*= *a_j _*- *a_i _*This shows the matrix is anti-symmetric.

#### BSM

Now we prove that there is a one to one correspondence between the *CSSM *matrices and the *BSM *matrices which is generated from the *CSSM *matrices. Suppose that (*A_i,j_*) is a CSSM matrix and the BSM matrix defined by

Now we show that

We know that *A_i,j _A_j,i _*< 0 or *A_i,j _A_j,i _*= 0. If *A_i,j _= A_j,i _*= 0, then *B_i,j _= B_j,i _*= 0 and the claim is true. If *A_i,j _*> 0, then *B_i,j _*= 4*A_i,j _*and *B_i,j _*= -2*A_j,i_*. This implies that

The case *A_i,j _*< 0 is similar.

### Descriptions of the biological data

**Figure 1:**
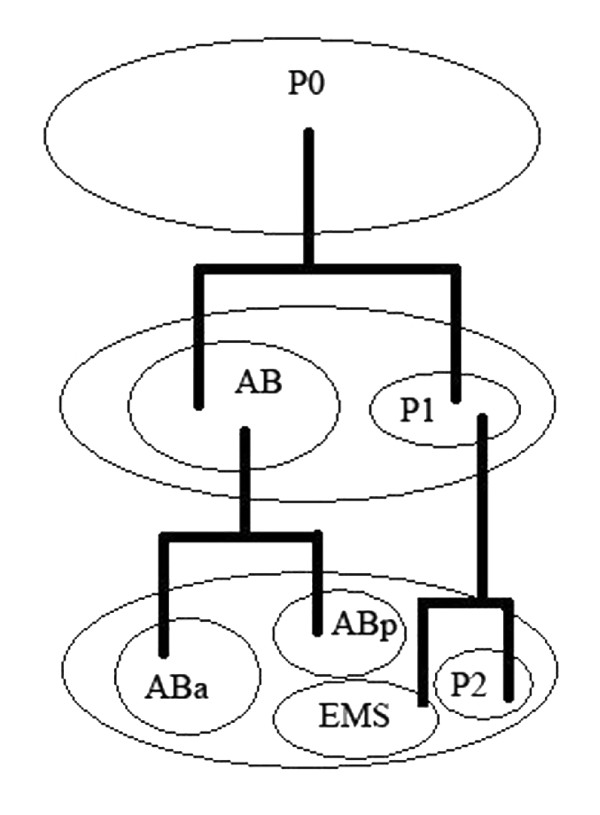
**presents the zygote (P0) at the 2-cell stage and the 4-cell stage of the early *C. elegans *embryogenesis**. The zygote divides into two cells, the anterior blastomere (AB) and the posterior blastomere (P1). AB divides into an anterior (ABa) and a posterior (ABp) cell, while P1 divides into EMS and P2. The long axis is formed by ABa and P2 and the short axis by ABp and EMS.

In the early stages of *C. elgans *embryogenesis, the zygote (P0) divides into two daughter cells called the anterior blastomere (AB) and the posterior blastomere (P1) forming a 2-cell stage embryo. This is followed by a second round of mitosis, where AB divides into ABa (anterior) and ABp (posterior), while P1 divides into P2 and EMS forming a 4-cell stage embryo (see Figure 1). ABp differentiates into different types of cells including neurons, body muscle, excretory duct cell and hypodermis, while EMS differentiates into 42 body muscles and intestine [63]. During organogenesis of the *C. elegans *embryo, ABp differentiates into a nervous system and epidermis, while EMS differentiates into muscular tissues, midgut and pharynx [64]. Axis determination is one of the most important events in the early stages of *C. elegans *embryogenesis; as the pronucleus breaks down in the zygote, asymmetric division follows forming a large daughter cell (AB) and a smaller one (P1) establishing the first antero-posterior axis. AB starts the next division, which is initially oriented orthogonally to the antero-posterior axis, but as the cell progresses through anaphase, the orientation of the mitotic spindle of the dividing cell skews, resulting in anterior position of ABa to ABp. P1 commences mitosis a few minutes later resulting in a large EMS progeny cell ventrally located, and a smaller P2 posteriorly located; this round of cell division establishes the dorso-ventral axis [65]. The other important event is garstulation, which begins at the 28-cell stage of development, where Ea and Ep move to the center of the developing embryo and gastrulate forming the three germ layers [64].

There are several reasons for comparing Abp-derived cells (ABp-dc) with those from EMS (EMS-dc). At the developmental level, ABp is derived from the AB blastomere while EMS is derived from the P1 blastomere [66], so ABp and EMS are two different lineages and the use of their cells is relevant in developmental biology. At the organogenesis level, ABp differentiates into nervous system and epidermis, while EMS differentiates into muscular tissue, midgut and pharynx [64], thus ABp and EMS form entirely different organs. At the cellular level, the cells derived from AB blastomere (ABa and ABp) enter the mitotic cycle and divide earlier than those of P1 (EMS and P2), while EMS enters the mitotic cycle earlier than P2 [67]. In addition, ABa and P2 align on the long axis (defining the anterior-posterior poles), while ABp and EMS align on the short axis (defining the dorsal-ventral poles) [68]. Therefore, from temporal and geometrical viewpoints, the derivatives of ABp and EMS are closer to each other, so a more powerful quantitative tool is needed to evaluate their development. At the molecular level, P2 is reported to induce polarization in ABp and EMS using MOM-2/Wnt signaling by direct contact between the cells [69].

### Experimental setting

The *C. elegans *data were obtained according to the previous report of Tiraihi and Tiraihi [70]. Briefly, a *C. elegans *embryo (from the 4-cell to the 80-cell stages) was considered, the Cartesian coordinates (*x,y,z*) were estimated from ABp and EMS cell lineages using the images obtained from SIMI-Biocell [71] and Angler softwares [72]. The Cartesian coordinates were entered into a computer program to calculate the distances between the cells.

The distances at 30, 55, 82, 109 and 123 minute intervals (fixed intervals) were used at different scales and the data were entered into a computer program used to calculate the zero centro-axial skew-symmetrical (CSSM) and the basic square matrices (BSM).

### Straight line

In the zero order straight line, two points represent the beginning and the end of the line. This line was divided into 48 unit lengths representing the steps (48 steps). The first order line was divided into two segments of equal length. At higher orders, each segment was divided into two equal parts, the box number increasing with the increase of order, leading to duplication in the number of the boxes and reduction in the step (see table 1). The SIPL method was applied to the straight line and the calculations were done as for the Koch curve (see below) except that the components of coordinates were taken from the straight line. The numbers of points at each order used in the study are presented in table 2.

**Table 1:** The box counting method data used in estimating the scale-invariant power law coefficient of the straight line.

Number of box (NB)	Logarithm (NB)	Step (h)	1/h	Logarithm (1/h)
1	0	48	0.020833333	-1.681241237

2	0.301029996	24	0.041666667	-1.380211242

4	0.602059991	12	0.083333333	-1.079181246

8	0.903089987	6	0.166666667	-0.77815125

16	1.204119983	3	0.333333333	-0.477121255

The box number (NB) and the steps (h) as well as the logarithm of NB and the logarithm of the inverse of step are presented. These data are plotted in figure 1, and the coefficients of the regression line were estimated in order to calculate the SIPL coefficient.

**Table 2:** The straight line orders and the related parameters used in calculating the scale-invariant power law coefficient using CSSM.

Order	# of Points at each order	Scale	Logarithm (Scale)	b-coefficient of linear regression	Logarithm of absolute b-coefficient
0	2	10^0^	0	-0.611244	-0.2137855

1	3	10^-1^	-1	-0.611244	-0.2137855

2	5	10^-2^	-2	-0.611244	-0.2137855

3	9	10^-3^	-3	-0.611244	-0.2137855

4	17	10^-4^	-4	-0.611244	-0.2137855

The orders, principal points and scales used in calculating the scale-invariant power law coefficient of the straight line using the zero-centro-axial skew-symmetrical matrix method and the b-coefficients of the regression lines are presented in this table. The data are plotted between the logarithms of the nth root of the absolute value of the basic square matrix at the order (where n is the number of principal points)  and the logarithms of the inverse of step (log(1/*h*)). The logarithms of the absolute b-coefficients are plotted against the logarithms of the scales. The calculations of the regression line of this plot were used in calculating the scale-invariant power law coefficient of the straight line.

### Koch curve

The zero order Koch curve is a line comprising two points at the beginning and end, which are named "initiator points". In order to generate a higher order Koch curve, every line segment was divided into three equal segments. We named the first and the third segments "resting segments" and the second a "generating segment". The resting segments stay unmodified, and as the name implies, generation takes place in the generating segment. For each line, if we build an equilateral triangle on the generating segment, the two new lines are called "generated segments". As the final step in generating the next order, we remove the generating segment(s).

In order to generate a CSSM, we need a set of Cartesian coordinates as the input. For every Koch curve, we consider the end points of every line segment. Before the generation of a higher order curve, the current points satisfying this criterion are called "resting points". After the generation, the newly generated points that satisfy this criterion are called "generated points". The ends of each line segment at a certain order are called that order's principal points.

The algorithm for generating a CSSM from a set of points was described earlier. The initiator line has two principal points, while the 1^st ^and 2^nd ^order Koch curve have 5 and 17 points, respectively.

**Figure 2:**
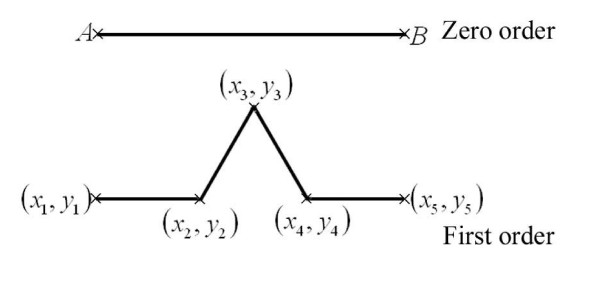
**presents two orders of a Koch curve**. The zero order is the initiator (straight line) with the initiator points (*A,B*); the components of the Cartesian coordinates of this order forming the (1 × n) matrix are (*x_a_*, *x_b_*) and (*y_a_*, *y_b_*). The first order Koch curve consisted of 5 principal points (2 initiators points (1 and 5) and 3 generated points (2, 3 and 4)); the components of the Cartesian coordinates of this order forming the (1 × *n*) matrix are (*x*_1_, *x*_2_, *x*_3_, *x*_4_, *x*_5_) and (y_1_, y_2_, y_3_, y_4_, y_5_).

A computer program was developed to generate the two-dimensional Cartesian coordinates of the points (as described above) of a Koch curve. In this program, the initiator line was a horizontal line of unit length, with the leftmost point (*A*) located at the origin and the rightmost point (*B*) at coordinates (1,0) (see Figure 2).

We can also scale the coordinate; for example the line is defined as (*A*(0,0), *B*(1,0))) at scale 10^0 ^and (*A*(0,0), *B*(10^1^,0))),(*A*(0,0, *B*(10^2^,0))), (*A*(0,0, *B*(10^3^,0))) (*A*(0,0, *B*(10^4^,0))) (*A*(0,0, *B*(10^5^,0))) and (*A*(0,0, *B*(10^6^,0))) at scales 10^-1^, 10^-2^, 10^-3^, 10^-4^, 10^-5 ^and 10^-6^, respectively.

The program calculated the components of the Cartesian coordinates of the principal points for the five orders at different selected scales. These principal points were entered into another algorithm in order to generate the zero centro-axial skew-symmetrical matrix (CSSM) and construct the basic square matrix (BSM) according to the method described in the appendix. The program calculated the two-dimensional Cartesian coordinates (*x,y*) at the different orders. In the first order (see Figure 2), the program calculated 5 principal points as (1 × *n*) matrices, hence there were five elements for the x (*x*_1_, *x*_2_, *x*_3_, *x*_4_, *x*_5_) and y (*y*_1_, *y*_2_, *y*_3_, *y*_4_, *y*_5_) components. These (1 × *n*) matrices (principal row) were used to generate 5 × 5 CSSMs and 5 × 5 BSMs. In the same way, for the other orders, the principal points of Koch curve were calculated and the principal rows and CSSMs were generated and the BSMs were constructed. If there were identical elements in the principal row, then one element would be included in the principal row and the others omitted, otherwise the constructed BSM of the generated CSSM would result in a singular matrix with zero determinant.

For example, for the first order matrix, the program calculated 5 principal points forming two (1 × *n*) matrices with 5 elements for each coordinate. This was also done for all the scales in the first order.

The data from the x-components of the Cartesian coordinates of the principal points (*A,B*) at zero order (initiator) of the Koch curve at 10^0 ^scale are (*A*(0,0), *B*(1,0))), the x-component of the initiator is [(*x*_a_, *x*_b_) = (0,1)] and the y-component is [(*y*_a_, *y*_b_) = (0,0)]. If(*x*_a_, *x*_b_) are considered as the elements of the (1, *n*) matrix, then the first row of this matrix is 0[1]. This was used to generate the CSSM for the x-components(*x*_a_):

where a stands for anti-symmetric.

The basic square matrix was constructed according to the algorithm presented in the appendix:

The determinant of this matrix is -8.

For the y coordinates, *y_a _*= 0 and *y_BSM _*= 0, the determinant of *y_BSM _*is zero.

Another CSSM was generated from the combined determinants of *x_BSM _*and *y_BSM_*; the input elements for the construction of this CSSM were (-8,0). It was translated to the point of origin to construct the CSSM; the principal row was [0,8]. The resulting matrix was:

And the resulting basic square matrix was:

The determinant of the basic square matrix  was -512, this value represents the determinant of the BSM at Koch curve segment level.

Five orders were used in the study (0^th^, 1^st^, 2^nd^, 3^rd ^and 4^th^), where the 0^th ^order is the initiator of Koch curve (straight line). Seven scales (10^0^, 10^-1^, 10^-2^, 10^-3^, 10^-4^, 10^-5 ^and 10^-6^) were used in the calculations. At each scale, the determinant of  was calculated.

Two main operations were involved in calculating the SIPL coefficient; the first was at the order level where *CSSM_order_*was used for subsequent calculations, while the second was at the scale level where linear regression was done in both. The first operation was subdivided into 3 sub-operations. In the first, an iterated algorithm was applied in order to generate *CSSM_order_*and construct *BSM_order_*. For the first order (see Figure 2), the determinant of  of the 0^th ^order was used as the first element of this matrix  and the second element was the determinant of the first order . Then the (1, *n*) of the first order to generate was . This was translated and used in generating *CSSM*_*order*(1)_, then *BSM*_*order*(1) _was constructed and its determinant det(*BSM*_*order*(1)_) was calculated. Similarly, for the second order, the (1, *n*) matrix was , which was used in generating *CSSM*_*order*(2)_, constructing *BSM*_*order*(2) _and calculating det(*BSM*_*order*(2)_). For the third and fourth orders (*CSSM*_*order*(3) _and *CSSM*_*order*(4)_), the (1, *n*) matrices were  and , respectively. Then *BSM*_*order*(3) _and *BSM*_*order*(4) _were constructed and their determinants, det(*BSM*_*order*(3)_) and det(*BSM*_*order*(4)_) were calculated. For the 0^th ^order,  was used for det(*BSM*_*order*(0)_). In the second sub-operation, the nth root of the absolute value of *BSM*_order _ (n is the number of principal points, where n = 2 in the initiator) was calculated. The calculations were repeated for all the scales (see table 1). In the third sub-operation, for a given scale, the data from the different orders at each scale were plotted using a log-log plot, where the abscissa was the logarithm of the inverse value of step (h) (log(1/*h*)), while the ordinate was the logarithm of the absolute value of . The log-log plot was fitted for linear regression and the b coefficients for the scales (*b_scale_*) were subsequently used in estimating the SIPL of the Koch curve.

For the next operation (scale level), the logarithm of the scale (log(*scale*)) was plotted against the logarithm of the absolute *b_scale _*and another linear regression was calculated. The b coefficient(*b_SIPL_*) was used in order to estimate the SIPL according to this equation: *SIPL *= 1- (*D*), where *SIPL *is the scale-invariant power law coefficient, and *D *is *b_SIPL_*.

### Assessment of validity

The diffusion-limited aggregate method was used to estimate the fractal dimension of the growing embryo according to Moatamed et al. [73]. Briefly, 5 concentric circles with 5 μm increments were superimposed on the center of gravity of ABp-derived cells (ABp-dc) and EMS-derived cells (EMS-dc) at the 123 min. stage of development, and the nuclei of the ABp-derived cells were counted within each circle. The log of the nuclear number was plotted against the log of circle radius, and the slope of the regression line was used as the value of the fractal dimension. The same procedure was done on EMS-dc.

### Programming languages

A computer program was written in the C++ language and a text file was generated containing the basic square matrix, which was copied into the command of the matrix of MATLAB^® ^software (http://www.mathworks.com: MathWorks, Inc, Natick, Massachusetts) and its determinant was calculated. Also, at each scale (10^0^, 10^-1^, 10^-2^, 10^-3^, 10^-4^, 10^-5 ^and 10^-6^), the determinants of the basic square matrices were calculated for the following Koch curve orders (0, 1, 2, 3 and 4). The step for each order was also estimated and entered into the calculations.

## Results

### Straight line

**Figure 3:**
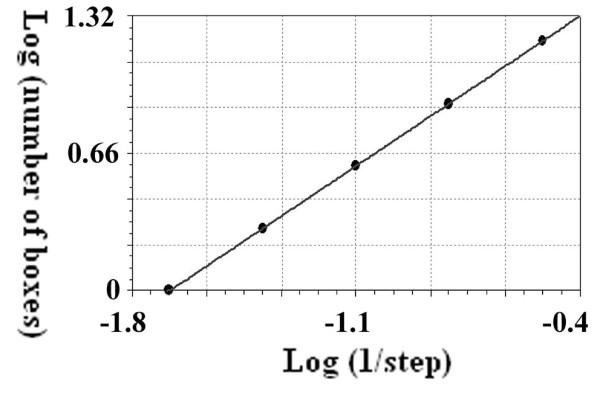
**Calculations of the scale-invariant power law coefficient of the straight line using the box counting method**. The latter is presented using Richardson's plot logarithm of the inverse of the steps plotted against the logarithm of the box numbers. The regression line has a slope equal to one (y = 1.7 + x: standard error = 0, correlation coefficient = 1).

The results for the straight line using the box counting method are presented in detail in table 1 while Figure 3 presents Richardson's plot; the slope of the regression line equals zero and the SIPL coefficient is one. Table 2 presents the data and the calculations for the straight line using the CSSM. It shows the 4 orders and the number of points at each order, the b coefficients of the linear regression at each order with different scales, the logarithm of the absolute value of b coefficients and the scales used for calculating the second regression line in order to estimate the SIPL using the b coefficients (D).

**Figure 4:**
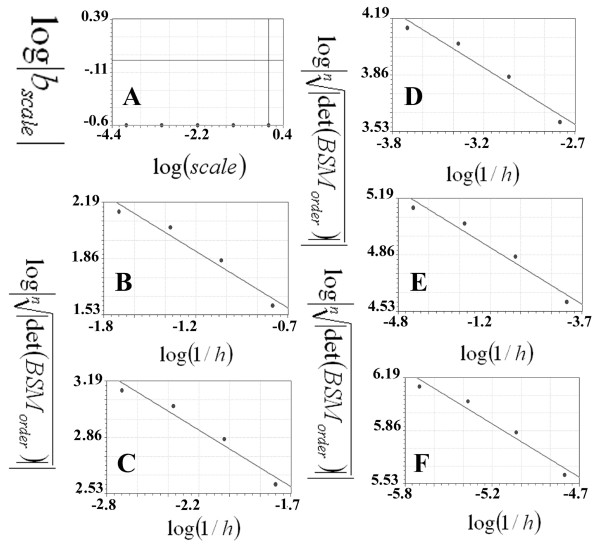
**Calculations of the scale-invariant power law coefficient of the straight line using the CSSM method; linear regressions were used to evaluate the straight line**. A: presents the logarithms of the scales (log(*scale*)) plotted against the logarithms of the absolute values of the b coefficients (log|*b*_scale_) in the regression line for the data plotted in this figure (B, C, D, E and F). The regression line has a slope equal to zero (y = -0.61: standard error = 0, correlation coefficient = 0.2). The linear regression of the logarithm of the inverse of the steps (log(1/*h*)) is plotted against the logarithm of the roots of the number of the points on the straight line to the absolute value of the determinant of the basic square matrix . Plot B: presents scale 1 (10^0^) unit, where the 0^th^, 1^st^, 2^nd^, 3^th ^and 4^th ^orders consist of 2, 3, 5, 9 and 17 points, respectively. C, D, E and F present the other four scales: 10^-1^, 10^-2^, 10^-3 ^and 10^-4^. The linear regression analyses of the plots are as follows: y = 1.15-0.61x (standard error = 0.061, correlation coefficient = 0.98), y = 1.54-0.61x (standard error = 0.061, correlation coefficient = 0.98), y = 1.93-0.61x, (standard error = 0.07, correlation coefficient = 0.98), y = 2.32-0.61x (standard error = 0.061, correlation coefficient = 0.98) and y = 2.7-0.61x (standard error = 0.06, correlation coefficient = 0.98) for plots A, B, C, D and E; the b coefficients of these regression lines represent (*b*_scale_).

Figure 4-A presents the logarithms of the scales (log(*scale*)) plotted against the logarithms of the absolute values of the b coefficients (log|*b_scale_*|) in the regression line for the data plotted in Figure 4-B, C, D, E and F, representing the scales 10^0^, 10^-1^, 10^-2^, 10^-3 ^and 10^-4^, respectively. The abscissa represents the logarithm of the inverse of the steps plotted against  (ordinate). In each plot, the 0^th^, 1^st^, 2^nd^, 3^rd ^and 4^th ^orders were entered in order to calculate the linear regression. The logarithms of the absolute values of the b coefficients of the above plots were used for estimating the SIPL of the straight line, which were plotted (ordinate) against the logarithm of the scales (abscissa). The SIPL was calculated as follows: *SIPL *= 1- (*D*), where (*D*) is the b coefficient of the regression line.

### Koch curve

**Figure 5:**
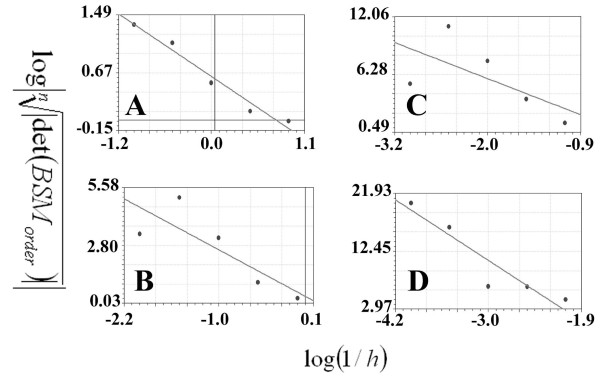
**Calculations of the scale-invariant power law coefficient of the Koch curve using the CSSM method**. The linear regression of the logarithm of the inverse of the steps (log(1/*h*)) is plotted against the logarithm of the roots of the number of the points on the Koch curve to the absolute value of the determinant of the basic square matrix . Plot A: presents scale 1 (10^0^), where the 0^th^, 1^st^, 2^nd^, 3^rd^, 4^th ^and 5^th ^orders consist of 2, 5, 17, 65, 257 and 1025 points, respectively. B, C and D present the other three scales: 10^-1^, 10^-2 ^and 10^-3^. The linear regression analyses of the plots are as follows: y = 0.58-0.777 x (standard error = 0.12, correlation coefficient = 0.98), y = 0.367-2.13 x (standard error = 1.23, correlation coefficient = 0.83), y = -0.577-3.153x (standard error = 3.24, correlation coefficient = 0.65, and y = -15.45-8.67x (standard error = 2.85, correlation coefficient = 0.93) for plots A, B, C and D.

**Figure 6:**
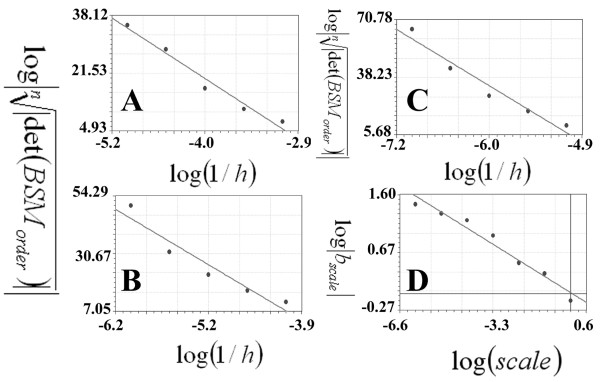
**Calculations of the scale-invariant power law coefficient of the Koch curve using the CSSM method**. The linear regression of the logarithm of the inverse of the steps (log(1/*h*)) is plotted against the logarithm of the root of the number of the points on the Koch curve to the absolute value of the determinant of the basic square matrix . Plot A: presents scale 4 (10^-4^) where the 0^th^, 1^st^, 2^nd^, 3^rd ^and 4^th ^orders consist of 2, 5, 17, 65, 257 and 1025 points, respectively. B and C present the other two scales: 10^-5 ^and 10^-6^. The linear regression analyses of the plots are as follows: y = -41.44-15.187x (standard error = 2.36, correlation coefficient = 0.98), y = -73.81-19.81x (standard error = 5.02, correlation coefficient = 0.96), y = -134.43-27.77x (standard error = 5.45 and correlation coefficient = 0.98) for the A, B and C plots. D: presents the logarithms of the scales (log(*scale*)) plotted against the logarithms of the absolute values of the b coefficients (log|*b_scale_|*) in the regression line for the data plotted in figure 3 (A, B, C and D). The regression line has a slope equal to zero as it is presented in a linear regression (y = 0.017-0.26x: standard error = 0.12, correlation coefficient = 0.98).

Figure 5 presents the first set of the linear regression of the Koch curve at different scales. There are 4 plots (A, B, C and D) related to 10^0^,10^-1^, 10^-2 ^and 10^-3^; three other plots are presented in (Figure 6A, B and 6C) representing the 10^-4^, 10^-5 ^and 10^-6 ^scales, respectively. The abscissa presents the logarithms of inverse of the steps (log(1/*h*)) which were plotted against  (ordinate). In each plot, the 0^th^, 1^st^, 2^nd^, 3^rd ^and 4^th ^orders were entered in order to calculate the linear regression; the b coefficients are presented in table 3. Figure 6-D presents the plot used for calculating the SIPL (*SIPL*) of the Koch curve, where the logarithms of the absolute values of the b coefficients(log|*b_scale_*|) of the above plots are plotted against the logarithms of the scales (log(*scale*)). The slope of the regression line was -0.25994438, while the SIPL was 1.25994438 calculated according to the equation SIPL = 1- (*D*). The analytical fractal value deviation from that was -0.15177%.

**Table 3:** Koch curve orders and the related parameters used in calculating the scale-invariant power law coefficient using CSSM.

Order	# of Points at each order	Scale	Logarithm (Scale)	b-coefficient of linear regression	Logarithm of absolute b-coefficient
0	2	10^-1^	-1	-2.1323166	0.328851688

1	5	10^-2^	-2	-3.1527369	0.49868773

2	17	10^-3^	-3	-8.6691842	0.937978231

3	65	10^-4^	-4	-15.187111	1.181475167

4	257	10^-5^	-5	-19.809067	1.296864021

5	1025	10^-6^	-6	-27.767092	1.44353039

The number of the orders, principal points and scales used in calculating the SIPL coefficient of the Koch curve using the zero centro-axial skew-symmetrical matrix method and the b-coefficients of the regression lines are presented in this table. The data are plotted between the logarithms of the nth root of the absolute value of the basic square matrix at the order (where n is the number of principal points)  and the logarithms of the inverse of step(log(1/*h*)). The logarithms of the absolute b-coefficients are plotted against the logarithms of the scales. The calculations of the regression line of this plot were used in calculating the scale-invariant power law coefficient of the Koch curve.

### Diffusion-limited aggregation

**Figure 7:**
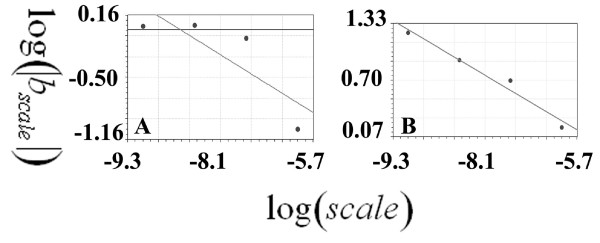
**The regression line of the fractal dimension calculated according to the diffusion-limited aggregate method in the ABp and EMS lineages**. This shows the plotted data of the log of the nuclear number against the log of the circle the radius; the b coefficient of the slope of the regression line represents the fractal dimension.

The fractal dimension of ABp-dc was 2.24 (standard error = 0.094; correlation coefficient = 0.99), and that of EMS-dc was 0.96 (standard error = 0.084; correlation coefficient = 0.96) (see Figure 7).

### Developing embryo

**Figure 8:**
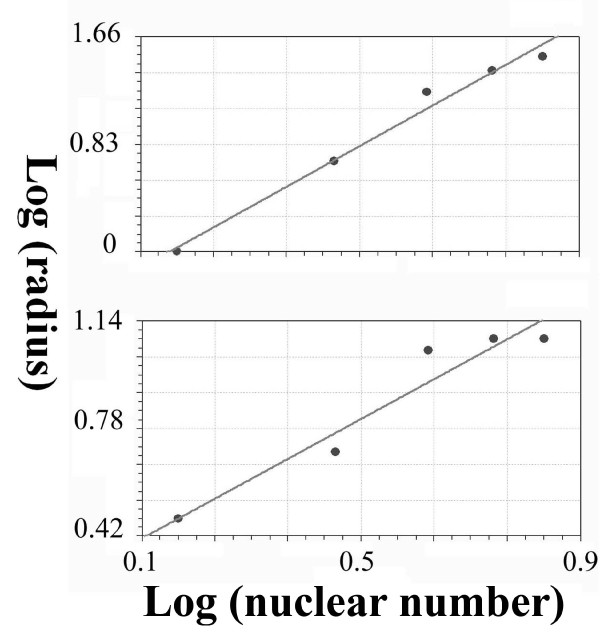
**shows the regression line of the plotted data of the scale-invariant power law coefficient in ABp and EMS lineages (one plot from each lineage as a sample)**. A: presents the scale-invariant power law coefficient in ABp. The b coefficient of another regression line between (log|*b_scale_|*) and the logarithm of the scale (log(*scale*))) was calculated, and the b coefficient of this regression line (*b_o_*) was estimated. *SIPL_o_*was calculated according to this equation: *SIPL_o _= 1-(D) *where D is *b_o_*. B: presents the plots of the scale-invariant power law coefficient calculations in the EMS lineage.

The SIPL coefficients were 1.34176236 and 1.33912941 in ABp-dc and EMS-dc, respectively; the regression line of the plotted data is presented in Figure 8.

## Discussion

The primary data used in the fractal analysis of the morphological studies are the lengths of the boundaries of the structure, which become more irregular with the increase of scale or magnification [74]. In this study, the components of the Cartesian coordinates were used as the primary data in the calculation of the SIPL coefficient. The development of an embryo from a fertilized egg is a dynamic process with space and time domains [75]. The spatio-temporal dynamics in developing embryos have studied by several investigators; for example, Wu et al. evaluated fetal development by plotting the fractal dimension of the brain surface at different stages of development against the time of brain development (in weeks) [76], and a similar approach was used by Schaffner and Ghesquiere in evaluating the complexity of type 1 astrotrocytes using the changes in the fractal dimensions during in vitro differentiation of the cells against the time of cell culture [77]. A different approach was taken to evaluating the changes in the value of the fractal dimension using it as a function of time [78]. While self-similarity is a property of fractals [79], cells in the developing *C. elegans *embryo migrate in a defined direction, becoming located in specific positions as they move. At each stage, the position of each cells is entirely different from the preceding and succeeding stages [70], so self-similarity does not hold during the development of the ABp and EMS sublineages of the *C. elegans *embryo. Fractal analysis was used in this study in order to confirm only the power law property of the SIPL, not self-similarity.

### The straight line and Koch curve

The SIPL coefficient of the straight line using this method was equal to the fractal dimension using the box counting method, which is consistent with the analytical value of the fractal dimension. The standard error of this regression line was zero. The SIPL of the Koch curve was 1.25994438 with standard error and correlation coefficient equal to 0.1214845 and 0.9810513, respectively. The deviation from the analytical fractal dimension value was -0.15177%. The box counting method was reported to be the most suitable and appropriate of the methods discussed by Mandelbrot for estimating the fractal dimension [80] and it is the most popular one [81]. The criterion for selecting the method is based on the consistency of the fractal dimensions of the straight line and Koch curve. Mola et al. used the box counting method to estimate the fractal dimension of a Koch curve [82], and the results deviated by 2%. Applying the box counting method in calculating the fractal dimension using the fractal dimension calculator [83] resulted in a deviation from its analytical value of 6%. Cajueiro et al. used an isometric grid and the result deviated by 0.25% from the analytical fractal value [84]. Jiang et al. also used the box counting method and reported that 3, 4 and 5 recursive calculations of the Koch curve resulted in fractal values of 1.212, 1.226 and 1.255 (3.8, 2.6 and 0.4% deviation from the analytical value: 1.26) [85], respectively. Therefore, the consistency of the SIPL coefficient with the fractal dimension suggests the power law property of SIPL. Moreover, a few studies have used the Cartesian coordinates as the primary data set in calculating the fractal dimension [86,87], and reported it to be difficult [88], though the use of Cartesian coordinates in field of morphogenesis has been reported [89]. Also, the application of different methods of fractal dimensions in spatial classification has been considered useful [44], but in the development of the embryo, the spatio-temporal events are more essential for dynamic studies [70].

### Diffusion-limited aggregation (DLA)

While diffusion-limited aggregation is used to study aggregation in clusters of particles in the time domain [79], morphogenesis in the developing embryo was studied using the DLA model, which confirmed the fractal property of the developing embryo [90]. DLA was suggested for quantifying the fractal dimension of blood vessel formation in the developing embryo [91] and other branching systems [92]. Therefore, it was used in this study in order to compare it with SIPL in the developing embryo. DLA was also used in the analysis of tumor vasculature [93,94], tumor growth [73,95-99] and in vitro tumor spheroids [100]. The emergence of complex morphology in developing organisms was reported to be caused by DLA [101]. An in vitro study of embryonic retinal neurons showed a decrease in the number of neurite branches with an increase in viscosity of the medium, which was interpreted as a DLA mechanism [102]. Vilela et al. reported that DLA could be used in describing biological growth processes [103]. Therefore, it was used in this study in order to verify the feasibility of using SIPL in the developing embryo.

DLA is a physical technique for describing the process of particle addition to a growing cluster of particles resulting in a power model for their number [79]. The fractal dimension calculated by DLA demonstrates the gradient of the diffusing substance toward the cluster [93]. Ryabov et al. reported that the gradient of these particles was proportional to the growth rate [104]. Therefore, the fractal dimension measured by the DLA method represents the growth rate, where the DLA value indicates that ABp grows more rapidly than EMS; this is also consistent with the SIPL results. Moreover, the value of DLA in ABp and EMS is different from the value estimated by SIPL, and the difference could be attributed to the feature of fractal dimension measured by DLA [105]. On the other hand, the large difference in values between ABp and EMS can be explained on the basis of the property of the DLA model. Peker et al. reported that two types of cluster formation resulted from DLA: the first underwent simultaneous aggregation-fragmentation processes or restructuring during growth; in the other type, growth depended on the immediate environment of the position of the new particles [96]. In the developing embryo, ABp-dc and EMS-dc can be considered as separate clusters, and the differentiating cells as particles. In previous studies, the results showed that EMS-DC had earlier regionalization, as the cells were tethered to neighboring cells by adhesion molecules [70], which is consistent with the second type of cluster formation. On the other hand, the cells (particles) of ABp-dc are separated into four groups, resulting in the spread of the cells in all directions (*x*, *y*, *z*), hence the value of the fractal dimension between 2 and 3 (which may indicate cell behavior similar to the first type of cluster formation) compared with that of EMS-dc (between 0 and 1), as the cells moved in one direction [70]. This analysis of ABp-dc is a consistent property of the fractal dimension of DLA, where the fractal dimension is a function of time, and the rate of aggregation is reduced as the cluster increases, as a result of spontaneous restructuring [106].

### The developing embryo

A qualitative study was done on self-organization based on the expression of PAR-3, PAR-6 and PKC-3 at the anterior pole of the *C. elegans *ovum with PAR-1 serine/threonine kinase and the PAR-2 proteins resulting in anterio-posterior polarization of the embryo [107]; also, there was self-organization at all biological hierarchy levels [1]. In fact, an in vitro study on gastrulation in isolated P1-descendent cells at the 26-cell stage of the *C. elegans *embryo (tracked in the cultured isolated cells using a 4D videomicroscope) showed that the cells gastrulate similarly to those of the intact embryo [108]. Also, a quantitative evaluation of the motion of the isolated cells using an in vitro setting showed that the onset of cellular motion was similar to that in the intact embryo (in vivo) and that the direction of the P1-descendent cells was also similar to that of the in vivo tracked cells [108]. This may indicate that patterning is required for cell population dynamics with the tendency of cells to associate with each other during gastrulation resulting in self-organization [49]. For example, in the vertebrate embryo, vascular patterning is essential for gastrulation movement [109]. On the other hand, in invertebrates, a quantitative study on video microscopy tracked cells at the early stages of *C. elegans *embryogenesis showed that their motion in the intact embryo was non-random in both EMS and ABp sublineages [70]. In this investigation, we showed that the SIPL coefficient of the EMS lineage is lower than that of the ABp, which is consistent with previous findings about the forward migration index (an index for chemotatic bias) [70]. Therefore, the lower value of the SIPL coefficient is not due to a decline in the complexity [26] or decomplexication of the sublineage [25], but shows that EMS-derived cells start to form an organized structure more rapidly as they tend to regionalize earlier than those of ABp [70]. Tabony documented that self-organization was not related to a single element such as the founder cells in the *C. elegans *embryo, but arose from the non-linear dynamics of all the elements collectively coupled to each other [110]. Schulze and Schierenberg revealed that embryonic cell lineages of low complexity formed a single sublineage or generated a single tissue type [111], as in the case of the E cell (from the EMS sublineage). Also, Quintana et al. reported that the isolated cells should adhere to each other in order to form an organized structure and suggested that a similar mechanism operated during the early stages of embryogenesis [12].

The results of this investigation are consistent with previous calculations of the diffusion coefficient, a physical parameter reported in a previous communication to be lower in the EMS lineage than in ABp during the early stages of embryogenesis in *C. elegans *[70]. Moskal and Payatakes reported that a decrease in the fractal dimension indicated a reduction in the diffusion coefficient, so the lower scale-invariant power law coefficient in the EMS lineage may indicate an overall slower motion of EMS-derived than Abp-derived cells. A reduction in the fractal dimension indicates a reduction in the Brownian diffusion coefficient [112], which possesses a random walk property [113], so the results indicate that ABp cells moved more randomly than those of EMS.

The values of the SIPL coefficients in ABp and EMS are 1.34176236 and 1.33912941, respectively (small difference), but these values were taken from logarithmic regression and their anti-logarithms are 21.97 and 21.83, respectively, which are higher than the original values.

## Conclusion

The study demonstrates that self-organization takes place during the early stages of embryogenesis, as confirmed by a scale-invariant power law method, calculated by using a centro-axial skew symmetrical matrix. The latter was generated from the components of the Cartesian coordinates. The SIPL coefficient results are consistent with DLA, where the fd calculated by DLA indicated that the cells in ABp-dc behaved similarly to type 1 cluster formation, while in EMS-dc they behaved similarly to that of type 2.

## List of abbreviations used

BSM: basic square matrix; CSSM: zero centro-axial skew-symmetrical matrix; dc: descendent cells, derived cells; det: determinant of a matrix; DLA: diffusion-limited aggregation; fd: fractal dimension; SIPL: scale-invariant power law.

## Competing interests

The authors declare that they have no competing interests.

## Authors' contributions

MT developed the software for generating the data of the components of the Cartesian coordinates and developed the computational techniques in the study, AT contributed to the data analysis and other calculations, and to the writing and organization of the paper. TT developed the theoretical framework, proposed computational techniques, guided the study and led the writing and organization of the paper. All authors read and approved the final manuscript.

## Appendix

The model used for evaluating the methodology has three points; each was assumed to be a center of gravity of a nucleus of a cell with two neighboring cells. The Cartesian coordinates of the cells were taken in two dimensions and the point of origin of the Cartesian coordinates was translated sequentially to the centers of gravity of all the cells. The x-and y-coordinates of the three cells were used for generating a(3 × 3) zero centro-axial skew-symmetric square matrix (CSSM). Another matrix, a basic square matrix (BSM), was constructed by multiplying the negative elements of the skew matrix with the negative operator (-1), while the positive elements were multiplied by the positive operator (+2). Then all the elements were multiplied by a scalar operator (+2) and the determinants of the matrices were calculated using Matlab software http://www.mathworks.com.

### CSSM generation

**Figure 9:**
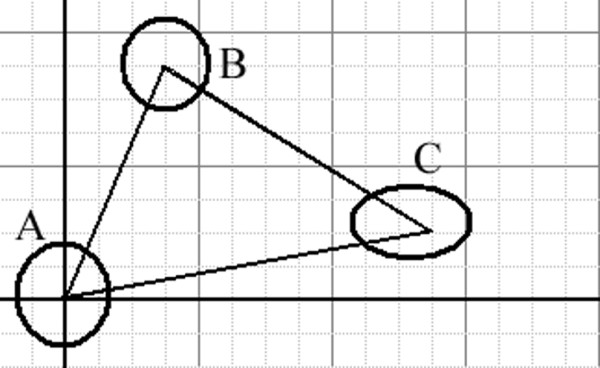
**The model presents the method for generating the zero centro-axial skew-symmetrical matrix**. This represents the schematic positions of the nuclei of three cells (A, B and C) in a two-dimensional space, where the center of gravity of cell A is located at the origin of the Cartesian coordinates. This was used to generate the array of the zero centro-axial skew-symmetrical matrix (CSSM) for both the x and y components.

There are two algorithms for generating CSSM. The first algorithm was accomplished according to the model shown in Figure 9. The nuclei of the three cells (A, B, C) can be seen. There are three states of translating the origin of the Cartesian coordinates (0, 0) to the center of the gravity of each cell:

State 1:

The origin of the Cartesian coordinates is located at the center of nucleus A, and the values of the coordinates for the cells are as follows:

The set of cells is (A, B, C) and the Cartesian coordinates are [(0, 0), (3, 7), (11, 2)]

State 2:

If we translate the origin of the Cartesian coordinates to the center of gravity of cell B, the values will be as follows:

The set of cells is (A, B, C) and the Cartesian coordinates are [(-3, -7), (0, 0), (8, -5)]

State 3:

Likewise for cell C:

The set of cells is (A, B, C) and the Cartesian coordinates are [(-11, -2), (-8, 5), (0, 0)]

Table 4 presents the arrangement of the x-components, while table 5 presents the y-components for the cells A, B and C in the three states. The array of numbers in table 4 can be presented in this skew matrix, which represents the x-component of the Cartesian coordinates of the centers of gravity of the nuclei in A, B and C cells:

**Table 4:** The array of the x-coordinates of the three states of translation for the three cells.

	Distance of the center of the gravity from cell		
TCC-CG of	A	B	C

A cell (x-coordinate)	0	3	11

B cell (x-coordinate)	-3	0	8

C cell (x-coordinate)	-11	-8	0

TCC-CG: translation of the origin of the Cartesian coordinates to the center of gravity of the nucleus of either A, B or C.

**Table 5:** The array of the y-coordinates of the three states of translation for the three cells.

	Distance of the center of the gravity from cell		
TCC-CG of	A	B	C

A cell (y-coordinate)	0	7	2

B cell (y-coordinate)	-7	0	-5

C cell (y-coordinate)	-2	5	0

TCC-CG: translation of the origin of the Cartesian coordinates to the center of gravity of the nucleus of either A, B or C.

Similarly, the array of the y-components in table 5 is presented in the following skew matrix:

This is a zero centro-axial skew-symmetrical matrix (CSSM).

The second algorithm for CSSM generation was done as described in the methods section. Initially, (1 × 3) matrices for the x-and the y-components [(0,3,11) and (0,7,2), respectively] were used for generating the skew matrices. The CSSM were generated:(1)(2)

Where *i *and *j *are indices for rows and columns, respectively.

In Euclidean space, the formula can be generalized into n-dimensional space where n = 1, 2, 3, 4.....N, and N is a finite number.(3)

According to Hadley's [114] notation, the skew symmetric matrices can be rewritten as follows:

and

where the "a" subscript stands for anti-symmetric.

2-Construction of the basic square matrix

The basic square matrices (*BSM*) *x_BSM _*and *y_BSM _*were constructed by multiplying the negative elements of the CSSM by operator (-2) and the positive element by (+4):

and

The determinants of the basic square matrices (and det(*y_BSM_*)) were 12672 and 3360, respectively.

A 2 × 2 CSSM can be generated from the values of det(*y*_BSM_) by arranging them into a (1 × 2) matrix in the form of [det(*x_BSM_*), det(*y_BSM_*)]. This matrix will then be input to the CSSM generation routine and a BSM will be constructed from the CSSM.

For example, for the above values, the input matrix to the CSSM generation process (as described above) will be [12672, 3360] and the CSSM will be (see table 6):

**Table 6:** The (2 × 2) array generated by translation of two points (det(*x_BSM_*) and (det(*y_BSM_*)) on the real axis to the origin of real axis.

Translation of point on origin of the real axis	det(*x_BSM_*)	det(*y_BSM_*)
det(*x_BSM_*)	0	-9312

det(*y_BSM_*)	9312	0

det(*x_BSM_*) is the determinant of the basic square matrix (BSM) constructed from the zero-centro-axial skew-symmetrical matrix (CSSM) presented in table 1.

det(*y_BSM_*) is the determinant of the basic square matrix (BSM) constructed from the zero centro-axial skew-symmetrical matrix (CSSM) presented in table 2.

and the BSM Matrix will be:

Finally, the determinant of this matrix is -693706752.
